# An Affective-Appraisal Approach for Parental Shared Decision Making in Children and Young People's Mental Health Settings: A Qualitative Study

**DOI:** 10.3389/fpsyt.2021.626848

**Published:** 2021-02-09

**Authors:** Shaun Liverpool, Daniel Hayes, Julian Edbrooke-Childs

**Affiliations:** ^1^Evidence Based Practice Unit, University College London and Anna Freud National Centre for Children and Families, London, United Kingdom; ^2^Faculty of Health, Social Care and Medicine, Edge Hill University, Ormskirk, United Kingdom

**Keywords:** parent, emotion, shared decision making, child, youth, mental health

## Abstract

**Background:** The majority of existing shared decision making (SDM) models are yet to explicitly account for emotion as an influencing factor to the SDM process. This study aimed to explore the role of parents' and carers' emotional experiences as a concept that has implications for SDM in children and young people's mental health (CYPMH) settings.

**Methods:** A social constructivist grounded theory approach, analyzing data from focus groups (*n* = 4) and semi-structured interviews (*n* = 33) with parents and healthcare professionals, was undertaken. Participants were identified and selected at CYPMH sites and through social media platforms or in-person advertising as part of a larger feasibility trial. Interviews and focus groups were audio-recorded and transcribed verbatim. Thematic analysis moved from open to focused coding.

**Results:** The majority of the sample consisted of mothers of adolescent girls. Healthcare professionals had an average of 7.54 (SD = 6.24) years of work experience in CYPMH outpatient capacities. Findings suggested that parents are “expected to, but not always able to” engage in SDM. Themes and subthemes described an affective-appraisal SDM process capturing: (1) views and experiences of SDM, (2) parents' emotional states, (3) the influence of emotions on SDM, and (4) key support systems accessed. The emerging affective-appraisal framework highlighted that negative emotional states hindered parents' active involvement in SDM, and positive emotions encouraged involvement in SDM.

**Conclusion:** The current findings describe an SDM model specific to CYPMH. This new understanding contributes to addressing a possible theory to practice gap opening new challenges and opportunities for academic enquiry.

## Introduction

Shared decision making (SDM) has been broadly defined as a cognitive, emotional, and relational process where service providers and service users collaborate to derive care and treatment decisions (1). Service user involvement in healthcare decisions is highly recommended, linked to better health outcomes and promotes satisfaction with services (2–4). In children and young people's mental health (CYPMH), service users include children and young people as primary service users and parents (including non-biological caregivers) as secondary service users (5). However, previous studies have mainly focused on the dyad relationships between service providers and primary service users (6). Therefore, the areas where triad relationships exist have been less understood; further highlighting the need for a consensus definition and unified view of SDM (6, 7).

To date, researchers have proposed several SDM models. The majority of the available models are embedded in adult healthcare, with fewer models specific to pediatric settings and mental health care (6, 8). Although some generic models propose that service users and their families should be a distinct and active part of the SDM team (9), this approach is yet to be taken up extensively in clinical care (10). In pediatric care, researchers highlight that the active participation from all parties (i.e., parent, child, and practitioner) is required for the decision making process to be regarded as SDM (11). However, other researchers accept the inclusion of the child, the caregiver, or both, with the possibility of including other stakeholders (12). These inconsistencies highlight a need to address gaps between theory and practice, suggesting further explorations to ensure appropriate decision-makers are included if implementing SDM in pediatric care (13, 14). Nonetheless, researchers agree that effective SDM requires active participation from service users (15). One such framework for youth SDM proposes (1) setting the stage for youth shared decision making, (2) facilitating youth shared decision making, and (3) supporting youth shared decision making (16).

Despite the efficacy of SDM, several barriers and facilitators have been identified and divided into categories of knowledge, attitudes, agreement, lack of expectancy or hope, and behaviors among service users and service providers (17). Reviews commonly highlight specific barriers such as patient/family characteristics (e.g., demographics and child health status), service constraints (e.g., time taken for consultation and trust in service providers), power imbalances, lack of available evidence-based treatment options, and service providers' limited knowledge of SDM skills (18, 19). In addition, researchers suggest that emotions may impact service users' involvement in SDM (20) and threaten parents' assumed role in the decision making process (21). Interviews with clinicians, parents and young people corroborated those findings, highlighting that strong emotional states affected the SDM process (22–24). The extant literature also highlights the need to increase awareness of emotions as social information influencing SDM (25). However, investigations of conversational interaction patterns in pediatric primary care indicated little opportunities of dialogue about emerging emotions from parents (26). As the Ottawa Decision Support Framework highlights the need for tailored decision support (27), further explorations could deepen our understanding of how emotional states influence the SDM process, with implications for intervention use (28). This approach also aligns with the Triangle of Care model which supports the working collaborations between service users, professionals and carers (29).

Although research investigating emotions and SDM is limited, many studies report heightened emotions in parents of children with mental health problems (30, 31). Previous qualitative studies in the United Kingdom (UK) broadly explored the emotions of parents of children with specific mental health disorders (32–35), belonging to specific minority populations (36), and of specific age groups below age 18 (37–40). The available research mainly focused on clinicians or therapists, and specific family groups. However, researchers also identified that around 20% of young people beyond age 18, especially those transitioning to adult mental health services, are supported by their parents (41). Thus, a research gap exists regarding the views of other healthcare professionals (HCPs) (e.g., nurses, support workers, medical social workers) and parents of children and young people (up to age 24) experiencing any mental health problem and accessing universal CYPMH care and treatment. In order to fully engage parents in SDM, it is important to understand the concept of SDM and how it applies to CYPMH care and treatment.

### Aims

This study has four primary aims. First, to provide insight into how HCPs and parents perceive SDM in CYPMH services. This understanding can inform and provide a common language for researchers to use when studying SDM in CYPMH. Second, to describe parents' experiences of SDM from the perspective of HCPs and parents. Third, to qualitatively explore emotion as an influencing factor for involvement in CYPMH decisions. Lastly, to identify parental support systems. This knowledge can inform the development of evidence-based decision support interventions and highlight the additional needs of decision-makers.

## Methods

### Research Team and Reflexivity

The interviews and focus groups were mainly conducted by the primary author. However, 2 focus groups were conducted by clinical researchers at the CYPMH site to maintain the privacy of the parents. The primary author has a background in health psychology, psychiatric research and policy development. The primary author was empowered, as a non-UK national, to ask neutral questions as there were no professional affiliations. The remaining authors have a background in CYPMH research and practice, and provided guidance throughout the study. The social constructivist approach accepts the researcher as part of the research process and therefore reflective journaling of thoughts were kept, and responses to data were discussed at regular meetings throughout data collection and analysis.

### Study Design

A social constructivist grounded theory approach was adopted (42). According to Charmaz (43), this approach allows the researcher to gain an insider perspective of the meanings of patterns of behavior that can be observed in a particular context. Thus, emerging concepts are socially constructed based on descriptions of the participants' experience of SDM. A qualitative study design, analyzing data from semi-structured interviews and focus groups of parents and HCPs was considered suitable to explore their views (i.e., beliefs and attitudes) and experiences of SDM (44).

### Study Settings

Participants were given the opportunity to choose between face-to-face interviews, phone interviews or in-person focus groups. Participants also had the opportunity to request the interviews be conducted at the CYPMH site or the university campus. These strategies were adopted to offer convenience and comfort and ensure privacy when conducting focus groups and interviews.

### Participant Identification and Selection

Parents were recruited from England in two strands: (1) as part of a feasibility and acceptability trial within the National Health Services (45), and (2) through social media platforms or in-person advertising. Parents were eligible if they (1) had at least one child or young person (0–24 years) with a mental health problem, (2) were over the age of 18, (3) had no known diagnosed mental health problems and (4) had the ability to speak and understand English. The young person's cut-off age at 24 years was selected as it coincides with the United Nations categorization of youth and young people (46).

Exclusion criteria were current involvement in any other research that had the potential to influence this study or if the child or young person was being treated under the Mental Health Act (1983). The Mental Health Act informs and influences how decisions are made for, with and about patients receiving mental health care in England. Parents were recruited through referrals from clinicians or self-referrals. Clinicians at the identified sites who participated in the feasibility trial relayed brief information about the study to the families, and parents who expressed interest were contacted by the site collaborator to be given further details about the study. After informed consent was obtained, the contact details of the parents were securely transferred to the research team. HCPs were also recruited as part of the feasibility trial. Information about the study was provided through presentations by the primary researcher at staff meetings. The site collaborators also identified and recruited HCPs. All staff working with families consisting of a child or young person experiencing mental health problems were eligible to be part of the study. Participants were contacted by email and/or phone call. If no answer was received, a reminder or follow up was sent a further 2 times, 1 week apart. If contact was unable to be made, participants were categorized as “unavailable.”

### Data Collection

Interview sessions (i.e., focus group discussions or individual interviews) were conducted between October 2018 and October 2019. Before the interview sessions, participants were briefed and informed consent was taken. Semi-structured interviews with open-ended questions were conducted. Probes were designed and utilized to generate further explanation from the participants without “leading” the interviewee. Interview guides were informed by previous research (47) and modified and refined to meet the aims of the current study. Questions focused on participants' views and experiences of SDM and how parents' emotions influenced the process (see Table 1). Interview schedules were used as a guide and there was freedom within the interview protocol to further explore some of the answers provided. The data were considered as saturated when the analysis did not produce any new concepts or further inform theory development (48). Interview sessions were audio-recorded and transcribed verbatim.

**Table 1:** Interview schedule.

**Questions (Healthcare professionals)**	**Questions (Parents)**
What does SDM mean to you?	What does SDM mean to you?
How do parents appear (i.e., emotionally) when engaging in SDM?	How do you feel (i.e., emotionally) when attempting to be part of SDM?
Is it important for parents to be part of the SDM process? Why?	Is being part of the SDM process important to you?
Where can parents access decision making support?	Where do you access decision making support?

### Sample Characteristics

Overall, data from *N* = 55 participants were included in the study. Four focus groups were conducted, *n* = 2 with parents and *n* = 2 with HCPs. The mean duration of the focus groups was 41.5 min, with an average of five participants. Additionally, 33 interviews with a total of *n* = 19 HCPs and *n* = 14 parents were conducted. The mean duration of the interviews was 26.2 min.

### Parents

Fourteen parents were interviewed and 10 participated in the focus groups. Of the total number of parents, there were *n* = 22 mothers and *n* = 2 fathers with a mean age of 44.88 (SD = 6.76) years. The majority of the parents identified as White British (95.83%) mothers, of girls (66.67%). The mean age of their children was 13.88 (SD = 2.8) years and experienced a range of parent-reported mental health problems (see Table 2).

**Table 2:** Characteristics of parents participating in interviews and focus group discussions.

**Variable**	**Interviews (*n* = 14)**	**FGDs (*n* = 2)**	**Total sample (*n* = 24)**
**Parent's age**			
Mean (SD)	45.93 (6.12)	43.4 (7.65)	44.88 (6.76)
Range	36–53	31–54	31–54
**Relationship to child** ***n*** **(%)**			
Mother	14 (100)	8 (80)	22 (91.67)
Father	0 (0)	2 (20)	2 (8.33)
**Ethnicity** ***n*** **(%)**			
White	14 (100)	9 (90)	23 (95.83)
Other	0 (0)	1 (10)	1 (4.17)
**CYP's age**			
Mean (SD)	14.36 (3.61)	13.2 (0.63)	13.88 (2.8)
Range	8–22	13–14	8–22
**CYP's gender** ***n*** **(%)**			
Male	5 (35.71)	2 (20)	7 (29.17)
Female	9 (64.29)	7 (70)	16 (66.67)
Other	0 (0)	1 (10)	1 (4.17)
^**a**^**CYP's clinical characteristics** ***n*** **(%)**			
^b^ADHD	1 (7.14)	0 (0)	1 (4.17)
Anxiety	0 (0)	4 (40)	4 (16.67)
^c^ASD	1 (7.14)	0 (0)	1 (4.17)
Depression	2 (14.29)	0 (0)	2 (8.33)
^d^PTSD	1 (7.14)	0 (0)	1 (4.17)
Comorbidities^*^	8 (57.14)	0 (0)	8 (33.33)
Undiagnosed^**^	1 (7.14)	6 (60)	7 (29.17)

* *Comorbidities included a subset of ADHD, Anxiety, ASD, Depression, self-harm, suicide attempt, psychosis, and Asperger's Syndrome*.

** *Undiagnosed represented children experiencing psychosocial difficulties but were not yet diagnosed*.

a Children or young people;

b Attention Deficit and Hyperactivity Disorders;

c Autism Spectrum Disorders;

d *Post-Traumatic Stress Disorders; SD, Standard deviation; FGD, Focus group discussion*.

### Healthcare Professionals

Nineteen HCPs were interviewed and 12 participated in the focus group discussions. HCPs represented a broad range of clinical expertise (e.g., Psychiatrist, Psychologist, Psychotherapist, Nurse, Occupational Therapist), worked with children and young people from ages 0 to 25 years in outpatient capacities and had an average of 7.54 (SD = 6.24) years of working experience (see Table 3).

**Table 3:** Characteristics of healthcare professionals participating in interviews and focus group discussions.

**Variable**	**Interviews (*n* = 19)**	**^a^FGDs (*n* = 2)**	**Total sample (*n* = 31)**
**Occupation** ***n*** **(%)**			
Consultant Psychiatrist	4 (21.05)	1 (8.33)	6 (19.35)
Psychologist/Psychotherapist	2 (10.53)	5 (41.67)	9 (29.03)
Nurse	2 (10.53)	4 (33.33)	6 (19.35)
Other^*^	11 (57.89)	2 (16.67)	10 (32.26)
**Clinical expertise** ***n*** **(%)**			
Eating disorders	2 (10.53)	0 (0)	2 (6.45)
General^**^	17 (89.47)	12 (100)	29 (93.55)
**Experience in CYPMHS**^**b**^ **(years)**			
Mean (SD)	6.36 (5.87)	9.40 (6.62)	7.54 (6.24)
Range	0.58–20	2.25–20	0.25–22

* *Other—represents Psychiatry/Medical Registrar, Occupational Therapist, Social Worker, Support Worker and Team Manager*.

** *General—working in general children and youth MH settings which includes, but not limited to, behavioral, attention deficit and autism spectrum disorders*.

a Focus group discussion;

b *Children and young people's mental health services*.

### Data Analysis

All transcripts were initially read in its entirety to obtain familiarity and an overall understanding of the contents. Interview transcripts were examined for more detailed descriptions of participants' views, and focus group discussions were examined for consensus or disagreement between participants. Data were analyzed using the thematic coding process outlined by Charmaz (43). More specifically, an iterative process consisting of open, axial and theoretical coding using inductive and deductive concepts was adopted. The first step generated initial codes from open coding in which units of meanings were derived from line-by-line analysis followed by axial coding to integrate and differentiate among subcategories. An independent investigator reviewed three random transcripts and generated codes. Codes were compared and discussed before inclusion. Theoretical coding was then used to identify relationships among categories. Demographic data and anonymous transcripts were linked and coded in NVivo 11 (49). Memos were written during the coding process to capture impressions and to facilitate interpretations.

### Ethical Approval and Trustworthiness

Ethical approvals were obtained from the London Surrey Research Ethics Committee (IRAS 236277) and University College London. The participants received both written and oral information about the study's purpose, confidentiality, voluntary participation and their right to terminate the interview at any point. Participants had access to this information at least 24 h before the interview sessions and were given the opportunity to ask any further questions before the start of the interview sessions. A relationship was established briefly with each interviewee before the interview. Reflective journaling of thoughts was kept, and responses to data were discussed throughout the study. At the point of analysis, weekly discussions occurred to explore emergent themes and achieve consensus. Additionally, member checking was done in the form of clarification probes throughout each interview to ensure the interviewer understood the information as the participant intended. The credibility was also enhanced by triangulation, collecting interview and focus group data from parents and HCPs who may have had different perspectives (50). Findings were reported according to the recommended guidelines for qualitative research (51).

## Results

The findings were organized according to the key research questions for this study. Responses were presented as categories of themes and subthemes (see Table 4). The following section highlights the themes and subthemes, reported using exemplary quotes with descriptive characteristics as labels.

**Table 4:** Summary of how the qualitative findings address the research questions.

**Research question**	**Categories of themes and subthemes**
How do parents and healthcare professionals describe SDM in current practice?	**Views and experiences** A somewhat collaborative process Positive experiences Negative experiences
What are parents' and healthcare professionals' views on the emotional experience of being involved in CYPMH decisions?	**Parents' emotional state** Positive emotions Negative emotions Mixed emotions
How do parents' emotional experiences impact on their involvement in the decision-making?	**Emotional influence** Facilitator or barrier
Where do parents access decision-making support?	**Support systems** Family's support network External agencies Online resources CYPMH site's internal resources

### How Do Parents and HCPs Describe SDM in Current Practice?

#### A Somewhat Collaborative Process

Generally, participants (when referring to both HCPs and parents) expressed an overall understanding that SDM was the “involvement” of key decision-makers in a process described as “collaborating,” “exchanging information” or “working together” to identify a care or treatment plan that was in the “best interest of the child.” Most participants were familiar with the concept and those who were unfamiliar were able to draw from their personal, lived experiences to describe SDM.

For me, I suppose shared decision making means some joined up thinking between clinicians, parents and young people if they're of an age where they can contribute and make their wishes known and their voices heard. (HCP, 13 years of experience)

Oh, it means sitting down together, discussing things, listening and then coming up with a plan. (Parent#1 of a 17-year-old)

Some participants expressed that the extent to which each decision-maker participated in SDM varied. The age and capacity of the child or young person and the nature of the decision were key factors to determine inclusion.

Erm.. Well depends on the sensitivity and age of the child because there are some things that I discuss, and I am not ok for my son to be around. (Parent of a 10-year-old)

Her dad would sometimes be part of it as well, but not all the time. So, it would be me alone or two or three of us and the clinician. (Parent of an 11-year-old)

Some participants also expressed that levels of involvement in SDM influenced who made the “final” decision. This suggested that at least one of the key decision-makers remains with the “final” decision making power. However, participants reported that the “final” decision generally occurred after the exchange of information and ideas. In some instances, it meant that a subset of the decision-makers was involved in the “final decision.”

Umm. I think it has been a mutual sort of everyone throwing ideas into the pot and then we kinda come up with a plan. The final decision is my daughters. (Parent#2 of a 17-year-old)

But it's not my decision, but I provide information so that they [parent and child] can make a decision. (HCP, 6.5 years of experience)

Despite the child or young person's age, participants generally expressed that it was important to include parents in the SDM process. Parents and HCPs stressed the importance of parents “being in the loop” and the impact on treatment outcome. However, it appeared that levels of involvement from parents also varied.

Not necessarily involved but informed is probably a better way to put it. Just to be informed as to what they were covering. Maybe what they'd advised her to try and do over the week. That kind of thing just to be more informed, I think. (Parent#1 of a 16-year old)

One, it gives the child a sense of they're not doing it alone, they've got somebody to go to who is informed and understands where they're going and what they've been through. If they're [parents] not involved, they [child] often feel very alone and in my experience, there's a lot of worse outcomes when the child is feeling alone. (HCP, 4 years of experience)

#### Positive Experiences of SDM

When SDM, as understood by the participants, occurred, it was mainly described as a positive experience. HCPs expressed the usefulness of SDM and how it helped facilitate the care and treatment process. They also valued the child's input and described it as very positive.

There are many occasions when a parent will not want a particular intervention. And the child is saying, “Actually, I think I do.” And the parent will support that child, even though they don't necessarily agree with it, which is heart-warming in a sense that they're giving the child the opportunity to express their own wishes. (HCP, 6.5 years of experience)

Personally I find it very useful because if you get the young person, the parents and clinicians all get together to target the same goal then I find it more successful, it's more likely the intervention works. (HCP, 1.5 years of experience)

Parents also found the experience of SDM very helpful. Some parents reported that this “shared” decision making also occurred outside of the medical encounter and was practiced within the family network. Therefore, experiencing SDM at CYPMH clinics was viewed as empowering and supported what one parent described as “interfamilial” decision making.

I think it's quite helpful. I think it's something that we generally did as a family anyway before my child became unwell in autumn last year. But I think we had, I don't know, lost the skill of that maybe by what had happened. And, so, it's been quite helpful and quite empowering and helpful that CAMHS have helped us to re-establish that, really. (Parent#2 of a 16-year-old)

#### Negative Experiences of SDM

There were more references made to negative experiences of not successfully achieving SDM on many occasions. It was expressed that the lack of available resources limited options and therefore, acted as a barrier to SDM. Shared decision making was viewed as appropriate when more than one choice was available. This was challenging for services, as service users were sometimes aware of additional resources that were not currently being offered by the clinics they attended, resulting in further disagreements. Similarly, disagreements existed between the parent and the child or young person on various topics (e.g., reasons for accessing service). HCPs expressed difficulty to manage these disagreements especially if the parents were not actively engaged. However, some parents felt that they were unable to provide input as they were unaware of the options.

Sometimes you just don't have any idea of what all this means, how do I know which would work and which would hurt her even more. I don't even know where to start or what's available (Parent of a 13-year-old)

…*there may not be much of a lay understanding about mental health within a family. So, when it comes to asking them what they think or what they might want etc., they really have no idea because they've not come across anything like mental health with their child or with any of their family members either. So, they really do then say, “Whatever you think is best, doctor.” So, I think that, obviously, makes shared decision making very hard. (HCP, 2.5 years of experience)*

### What Are Parents' and HCPs' Views on the Parents' Emotional Experience of Being Involved in CYPMH Decisions?

Parents identified a broad range of positive and negative emotional experiences. Similarly, HCPs described a broad range of emotions observed in the parents they encountered in routine care. These emotions (e.g., anger, stress, frustration, relief) were described on a spectrum.

Well, it can be a massive range; some are relieved, some are frustrated, some maybe angry, some are just really grateful that they're being seen. It just goes from one extreme to the other. It depends on the person and from the family of the young person's personal experience of being in the service. (HCP, 20 years of experience)

It always makes me feel quite anxious. Because I know that it makes my daughter then quite anxious and upset. She doesn't like talking about her problems. But it also makes me feel like I'm relieving something. (Parent of a 9-year-old)

#### Positive Emotions

Participants described positive emotions arising after a challenging period. Some parents described feeling a sense of relief of finally receiving a diagnosis or finally getting seen at CYPMH clinics. Additionally, after seeing their child “struggle” with mental health difficulties, parents expressed joy in seeing a positive outcome from treatment decisions or being able to share the burden.

It is more a sense of relief and being a bit more hopeful by the time they finish the session. (HCP, 10 years of experience)

…*after I understood what he is going through, or what I can do to help him, it became much, much less stressful. And in general, I am very happy with him and I don't have much stress anymore. (Parent#1 of a 14-year-old)*

#### Negative Emotions

On the other end of the spectrum, parents experienced emotions such as anxiety, worry, anger, frustration and fear. These feelings were also reported as being observed by HCPs in most cases, and participants reported that these emotions varied among families and situations.

I see a lot of frustration. Sometimes a lot of anger from the young people's families about the time that they've had to wait for specific treatments. (HCP, 1 year of experience)

I thought the world had stopped. This came like a bolt out of the blue, and for the first two days I didn't know what had hit me. I was absolutely shell shocked. (Parent#2 of a 14-year-old)

#### Mixed Emotions

Parents also described emotions as co-occurring or described having “mixed” feelings. Parents reported having to focus on the outcome of the decision and therefore, despite experiencing negative emotions, they felt a need to be involved. This conflict within themselves resulted in positive and negative feelings co-occurring. To illustrate, one parent stated,

Erm. Very mixed emotions. I mean you would rather not be in those decisions at all. But when you are in that situation, I am glad that she wants me there, I am glad that she wants me to support her and I am very glad that I have some idea of what is going on so I can support her more effectively. Umm I mean all of us are highly anxious. The anxiety of worrying about the wellbeing of my child. You got the anxiety at the initial sessions of what are these people thinking of you. There are lots of lots of feelings to be anxious but you manage it because you have to. (Parent#1 of a 17-year-old)

### How Parents' Emotional Experiences Impact Their Involvement in the SDM Process?

#### Facilitator or Barrier

Participants expressed that emotions generally influenced parents' involvement in care and treatment decisions. In some instances, they described the reverse also occurred where the involvement also affected the parents' emotional state. They expressed that both negative and positive emotions influenced involvement. More expectedly, negative emotional states resulted in parents not being actively involved in SDM and positive emotions encouraged involvement. Participants highlighted that in some instances, the negative emotions appeared to complicate the SDM process as it made it difficult to participate even if they wanted to. However, participants also expressed that negative emotions made some parents more “forceful” suggesting a form of over-involvement. Similarly, some positive emotions, like when parents were comfortable or fully trusting of the HCPs, they decided to be less involved. Other emotions such as relief, content, satisfaction and hope had a more positive impact on the SDM process and appeared to encourage parents to be actively involved.

If you're [parents] anxious and distressed, the anxiety may want you to kind of take full control and therefore, you're [parents] going to want to be more involved. But it might make them [parents] back off, so they might not want to be involved. However, if they've got that feeling of hope, because they think that they're in a position where I'm [HCP] talking like I know what I'm on about, then they may think, ‘All right, the doctor knows; I don't need to be so involved.' (HCP, 2.5 years of experience)

It was a very difficult and very stressful time. I think I was pretty passive at that time, yes. I wanted other people to tell us what was the right way to go to make life better for my daughter. Yeah. (Parent#1 of a 16-year-old)

### Where Do Parents Access Support?

Participants reported accessing various sources of support during decision making periods. Parents generally appreciated contact with and support from the family's own support network, external agencies, the CYPMH site and online services. Emotional support and knowledge support appeared to almost be used interchangeably. Although, family members and friends offered emotional support, in some instances, parents relied on their decision making input. Strategies that were described as “helpful” or “useful” varied in the participants' responses. The majority of HCPs referred parents to more than one resource, and many parents reported accessing multiple sources of support.

#### Families' Own Support Network

The support the parents needed and received from others varied between parents, over time and decision type. Many received support from family members, friends, and other parents. In some instances, parents received support from extended family members, e.g., grandmothers. In other instances, they described relying on support only between parent (s) and child.

Obviously, my husband. He's always my first port of call really with things like that. And then outside of that, friends and family. (Parent#2 of a 16-year-old)

Just my wife. (Parent of 12-year-old)

#### External Agencies

Parents reported accessing charities and other services for support. This was both practical (e.g., financial, information) and instrumental (e.g., seeking advice from persons with similar experiences). HCPs also reported referring parents to known charities and other support services.

We often refer them to the Early Help Hub, but they're kind of like a signposting service and they can access family therapy and family support workers. That's something I've done a couple of times recently. (HCP, 4 years of experiences)

#### Online Resources

The majority of HCPs reported signposting parents to online resources from “trustworthy” sources. There were some concerns from HCPs about parents using “Dr. Google” and encountering inaccurate or worrying information. However, parents admitted to using a wide variety of online websites and resources to gather information.

I use a few websites that are useful. I can always just use the internet and if I put in the right thing to search, I get a bit of information. (Parent of an 11-year-old)

#### CYPMH Site's Internal Resources

Generally, the CYPMH site was seen as a vital resource. Although some parents described the help as being solely for the children and young people, parents appreciated this as they felt happy knowing their child was being seen. However, HCPs reported having to spend time responding to parents' concerns outside of appointments. Interventions offered by the CYPMH services were limited but included interventions such as information outlets, signposting, parent groups and family therapy. When reporting family therapy and parent groups as sources of support, parents described shortcomings such as long waiting times and lack of time to attend group sessions.

That's probably the one downside is that my husband and my daughter are both on the list for family therapy, but the waiting list is so long I don't know when that's going to happen. (Parent#3 of a 16-year-old)

But on paper, we have family therapy, but it's pretty hard to get because of the waiting list. I think we have maybe one or two teams in our service that I'm aware of. But again, not enough service in my locality. (HCP, 4 years of experience)

### Summary of the Findings and Interviewer's Reflection

**Figure 1:**
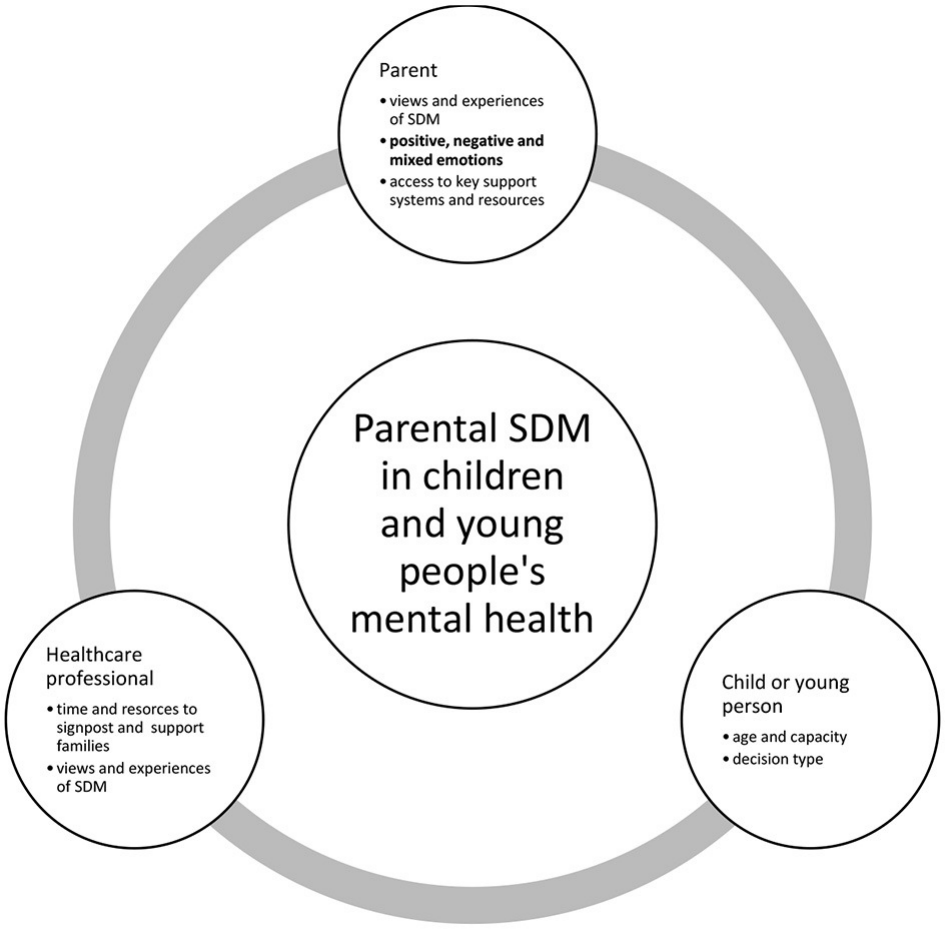
Conceptual framework of an emerging affective-appraisal model of parental involvement in SDM in children and young people's mental health.

An overall concept suggesting that parents are “expected to, but not always able to” engage in SDM encapsulates the findings. Themes and subthemes described: (1) views and experiences of SDM, (2) parents' emotional states, (3) the influence of emotions on SDM and (4) support systems accessed. The overarching themes were organized into a conceptual framework illustrating an evidence-informed affective-appraisal model of SDM (see Figure 1) in CYPMH. The figure depicts the key decision making actors and influencing factors. The affective-appraisal approach to SDM recognizes that affect and appraisal interact in shaping the SDM process, influencing each other in a circular way where the decision may elicit the emotional reaction, that in turn influences the SDM process, that again may influence a change in the emotional reaction. The findings suggest that adequately supporting parents can activate them to engage in high quality SDM. In this way, emotional support would allow the identification of parents' values and needs associated with SDM, thus enriching the SDM experience.

## Discussion

This study provided insight into the experiences of parents involved in CYPMH care and treatment decisions from the perspective of both HCPs and parents. The overarching concept illuminated the affective-appraisal approach framework to SDM that revolved around an interactive parent, child and HCP SDM process. In line with existing evidence, the appraisal process referred to ongoing value-based judgments linking emotion and cognition occurring before, during and after SDM. The current findings also agree with other researchers highlighting emotions as an influential factor to SDM (25). It was also observed that the circular framework replicated the triangular configuration in previous studies that emphasizes the dual role of parents as both services users and caregivers of their children (26). This study adds that decision maker's views and emotional experiences of SDM and access to key support systems appear to be essential to SDM in CYPMH settings.

The understanding of SDM from parents and HCPs also aligns with the extant literature on definitions of SDM as a collaborative process between service users and service providers (1, 7). However, our findings confirm the uniqueness of the triad in CYPMH decision making and may disagree with other researchers. Although Park and Cho (11) suggested that parents, children and HCPs should be involved for the process to be referred to as SDM, the current findings indicate that not all decisions makers need to be actively involved at all times for SDM to occur. Instead the current findings align with other researchers (12) and further highlights that the levels of participation in SDM may vary in different aspects of the process depending on the legal context, capacity, experience and expertise of the participants and type of decision. Therefore, further investigations are needed to identify if existing SDM measurements are accurately capturing the levels of involvement taking into account the “informed” vs. “involved” approach to SDM in CYPMH settings. This study also confirms there may be a lack of knowledge on SDM involving caregivers, especially when the primary service user is a child or young person (5, 15). Existing models discuss “shared decisions” which were not clear in the current findings as some participants stated that there exists a “final” decision-maker (s) at the end of the SDM process. This understanding suggested that the “final” decision may not be viewed as the end product of the SDM process, but further steps such as agreeing on the final decision (outcome) could be explored and may be unique to the field of child health. Having HCPs and parents explicitly agreeing with a child's or young person's choice of treatment may be empowering. This study also adds to the youth SDM framework (16) by highlighting the importance of identifying parents' and children's preferences for involvement when setting the stage for SDM, and capacity when facilitating SDM. The findings also build on that of Crickard et al. (16) by identifying specific sources of information for supporting youth SDM.

This study also extends on what is already known about the “emotional roller coaster” that parents of children with mental health difficulties experience (30, 31), suggesting implications for an effective SDM process. Although the current findings align with previous research identifying parents' emotions as a possible influencing factor to the SDM process (22, 24); the current findings, build on this knowledge by identifying positive, negative and mixed emotions as barriers or facilitators. Further to this, the current findings suggest a two-way direction that emotions may be influencing parents' involvement in SDM and vice versa. This supports theories in the cognitive literature around decision making and emotions highlighting that health decision making is challenging during emotional periods (52). Similarly, decision making under stressful conditions was proven to be difficult for parents in both quantitative and qualitative studies (53, 54). However, some parents in this study expressed having to “get on with it” despite their own personal feelings. This raises further questions around active and effective involvement. In that light, the current findings support previous research highlighting the expectation that parents are to be involved in the SDM process despite their emotional states (26). As a result, policy-makers, researchers, practitioners and families should work together to develop and promote support mechanisms that are suitable and effective in this population. Nonetheless, it is not yet clear why emotional states vary among different populations and at different times and therefore, future studies could further explore this phenomenon.

This study also highlighted that parents relied on additional support from service providers, and therefore, HCPs had to invest time to offer the necessary support to parents. CYPMH services mainly provide services for children and young people, and limited resources are available within services to support parents (41). Therefore, having interventions that can be used outside of regular appointments can impact both HCPs and parents. Many HCPs reported signposting parents to external agencies and websites, and parents themselves reported accessing charities and online services. The latter is in line with the help-seeking literature that suggests carers are increasingly seeking information from online resources (55, 56) and further highlights the relative importance as expressed by both HCPs and parents. Therefore, policy-makers and practitioners should take note as poor quality information may exist online and some external agencies may not follow appropriate ethical and practice guidelines (57). An exploration and standardization of the role the internet and external agencies play in providing information or added emotional support to parents are warranted so services can harness these resources as tools.

### Relevance to Clinical Practice and Policy

An efficient SDM process may help minimize frustrations and anxiety around care and treatment options. Although, HCPs and parents expressed positive experiences when involved in SDM, the perspectives of children and young people are critical before recommendations are made about who should be involved in the SDM process. Therefore, this study also highlighted that the triad should explore each other's preference for the level of involvement considering “informed” vs. “actively involved.” This approach can help further minimize the burden and anxieties parents face when being the sole decision-maker (53). If parents are able to share this responsibility in a “trusting” relationship while feeling listened to, this may positively influence the SDM process. Additionally, encouraging a wider partnership with schools and organizations can help support the SDM process by providing families with both information and emotional support.

The findings of this study could further inform the Triangle of Care model by highlighting the lack of or limited support for parents accessing CYPMH services. Although the child is viewed as the primary service user, the importance of parent involvement in the decision making was crucial for successful care and treatment. Therefore, increasing the time spent per client may allow time for HCPs to include and involve parents in the care and treatment plans, depending on the age and capacity of the child. Alternatively, implementing additional programs to support parents throughout crucial decision making time points may help improve experiences of SDM. Whilst this may be useful, HCPs and community services will need to be kept up-to-date with available resources. Lastly, it was noted that parents often access charities and other services outside of CYPMH services to receive the necessary support. Therefore, it would be recommended that policy guidelines are in place to provide a bridge between the community and CYPMH services to ensure consistency, competence and ethics are maintained.

### Future Directions

The affective-appraisal approach to SDM model provides a preliminary framework for future works. Therefore, the proposed framework is subject to further revisions and adaptations. First, it is critical to add the voices of children and young people to those of the parents and HCPs obtained in this study to provide an accurate perspective of SDM and the influence of emotions in CYPMH services. Second, a consultation exercise with SDM experts will be beneficial to further enrich our current understanding and interpretations. Third, although it may be challenging, it is important to empirically test the model, specifically controlling for sources of emotions (e.g., receiving a diagnosis).

It is also important to carry out program and intervention evaluations to identify and evaluate currently existing SDM support tools to identify which resources are most beneficial. The theory of “parents being expected to, but not always able to” be involved in CYPMH care and treatment decisions suggests that it would be of great value to continue to develop and implement SDM interventions to promote collaborative decision making. As the theory's transferability is strengthened by this study, the theory can be the basis for intervention development and future research. Therefore, adopting an affective-appraisal approach to SDM may help inform interventions and support families that are in need of additional support. Finally, a quantitative exploration informed by the grounded theory identified in this study may help develop inferences around group differences. This is especially important to ensure traditionally underserved and underrepresented families are targeted.

### Strengths and Limitations

This study included a large sample size allowing for the attainment of data saturation. This study also highlighted the views and experiences of parents of children of varying ages and experiencing a range of mental health problems. In addition, HCPs with a variation in clinical backgrounds were involved in this study, allowing for a much broader understanding of the field, as well as increasing prospects for the transferability of the findings. Another strength included the approach to explicitly consider emotion (or affect) and SDM, as well as the specific focus within services for children and young people. However, this study is not devoid of limitations. First, the majority of the sample (*n* = 51) was recruited through referrals from various CYPMH sites as part of a larger feasibility trial and therefore the researcher had limited control over who were invited and recruited into the study. It is possible that parents and HCPs who are more inclined to be involved in SDM may have expressed interest and therefore biased the study sample. However, high levels of involvement with SDM may have increased and maintained active discussion in the interview sessions. Second, the participants' characteristics were not matched to their contributions for the purpose of examining any potential variability among the participants. Similarly no major comparisons were made between participants' views on SDM and their actual experiences of SDM for the purpose of analysis. Therefore, experiences of SDM may be specific to the majority sample (i.e., White British mothers of adolescent girls) and caution should be taken when interpreting and deriving implications from these findings. This could be viewed as a major limitation if attempts are made to generalize the findings to other groups, as the experiences of parents may differ depending on the child's age and gender. Nonetheless, some variety may have existed in terms of child's symptoms, parent's age and HCP's experience that reflected multiple perspectives and a multidisciplinary view on SDM. Although the methods adopted was in line with the social constructivism approach promoting the infusion of understandings through social interaction, the current study acknowledges that the emotional state of HCPs and young service users were not reflected in the analysis. This limits the extent of interpretations as to how much parental emotions could have been influenced by others. In addition, participants were asked to discuss their experiences of SDM in CYPMH services. Due to the slight variations in how the participants defined SDM and the subjective nature of the question, participants may have selected to express their first or most recent recall of SDM, therefore neglecting other instances of SDM or lack thereof. Lastly, the perspectives of children and young people were not captured in this study, and therefore limits the extent which our findings can be readily incorporated.

## Conclusion

Previous research findings indicate that the involvement of parents in CYPMH is linked to better health outcomes. Although SDM is recognized as a person-centered approach for quality healthcare, this current study suggests that levels of involvement in SDM may vary and parents experience a spectrum of emotions that may influence their participation in SDM. Therefore, the importance of the SDM process in CYPMH cannot be underestimated, and SDM should continue to be assessed and supported. In particular, an affective-appraisal approach to SDM may be needed to adequately support parents. Future studies should continue to investigate this phenomenon.

## Data Availability Statement

The datasets generated in this article are not readily available because The data contains confidential information about the participants. Requests to access the datasets should be directed to shaun.liverpool.14@ucl.ac.uk.

## Ethics Statement

The studies involving human participants were reviewed and approved by Ethical Approvals were obtained from the London Surrey Research Ethics Committee (IRAS 236277) and University College London. The patients/participants provided their written informed consent to participate in this study.

## Author Contributions

Analysis and interpretation of the data was conducted by the SL and supported by the DH and JE-C. SL drafted the manuscript. DH and JE-C contributed to the editing and refinement of the article before submission. All authors contributed to the conception and design of the study.

## Conflict of Interest

The authors declare that the research was conducted in the absence of any commercial or financial relationships that could be construed as a potential conflict of interest.

## Abbreviations

CYPMH: Children and young people's mental health
HCP: Healthcare professional
SDM: Shared decision making
UK: United Kingdom.
